# Look into my eyes: a “faceless” avatar interviewer lowers reporting threshold for adult eyewitnesses

**DOI:** 10.3758/s13421-023-01424-4

**Published:** 2023-04-18

**Authors:** Che-Wei Hsu, Julien Gross, Marea Colombo, Harlene Hayne

**Affiliations:** 1 Department of Psychology, University of Otago, Dunedin, New Zealand; 2 Department of Psychological Medicine, University of Otago, PO Box 54, Dunedin, 9054 New Zealand; 3 School of Population Health, Curtin University, Perth, Australia

**Keywords:** Computer-mediated interview, Avatar interview, Eyewitness, Cognitive load, Gaze aversion

## Abstract

Evidential interviewing is often used to gather important information, which can determine the outcome of a criminal case. An interviewer’s facial features, however, may impact reporting during this task. Here, we investigated adults’ interview performance using a novel tool—a faceless avatar interviewer—designed to minimize the impact of an interviewer’s visual communication signals, potentially enhancing memory performance. Adults were interviewed about the details of a video by (1) a human-appearing avatar or a human interviewer (Experiment 1; *N* = 105) or (2) a human-appearing avatar or a faceless avatar interviewer (Experiment 2; *N* = 109). Participants assigned to the avatar interviewer condition were (1) asked whether they thought the interviewer was either computer or human operated (Experiment 1) or (2) explicitly told that the interviewer was either computer or human operated (Experiment 2). Adults’ memory performance was statistically equivalent when they were interviewed by a human-appearing avatar or a human interviewer, but, relative to the human-appearing avatar, adults who were interviewed by a faceless avatar reported more correct (but also incorrect) details in response to free-recall questions. Participants who indicated that the avatar interviewer was computer operated—as opposed to human operated—provided more accurate memory reports, but specifically telling participants that the avatar was computer operated or human operated had no influence on their memory reports. The present study introduced a novel interviewing tool and highlighted the possible cognitive and social influences of an interviewer’s facial features on adults’ report of a witnessed event.

Evidential interviewing is often used to gather important information from a witness or a victim, and the outcome of a criminal case can often hinge on the quality of the interview (Euale & Turtle, 1998; Yeschke, 2003). Interviews typically occur in a social setting that involves an interviewer, a witness, and an interaction between them. Interviewers’ nonverbal communication signals—including eye gaze, gestures, and facial expressions—may impede witnesses’ reports of witnessed events, in part, due to (1) increased distraction and (2) the interviewee’s perception of being socially evaluated (Doherty-Sneddon & Phelps, 2005; Glenberg et al., 1998). Given this, researchers have explored ways to minimize visual communication noise during investigative interviews to maximize the quality and quantity of eyewitness testimony. In the present study, we explored the effects of a novel interviewing tool in the form of a (faceless) avatar interviewer. Such avatars may help to minimize visual communication signals, thereby increasing memory performance during an interview about a prior event.

## Impact of distractions on cognitive tasks

Within a broader framework of attentional control theory, Glenberg et al. (1998, p. 651) described the adaptive function of selective attention for human survival. They argued that, given that our environments consist of myriad stimuli, we must engage in selective attention to help us focus on and process important features and filter out less relevant ones. In a social setting, like that of an investigative interview, we often engage with the interviewer’s feedback, including their eye gaze, facial expressions, and gestures. Although these signals can help us to gather nonverbal information to guide our responses, they can also be a distraction from the task at hand (Broaders & Goldin-Meadow, 2010). According to cognitive load theory (Sweller et al., 1998), attention is a limited cognitive resource, and attending to visual feedback signals can interfere with cognitive processes such as memory retrieval (Engstrom et al., 2013). This interference is particularly prominent when individuals carry out complex cognitive tasks (Kleider-Offutt et al., 2016; Sweller, 1988, 1994; Sweller et al., 1998). High cognitive load restricts information processing of relevant information (Van Merrienboer & Sweller, 2010), which in turn, hinders task performance (Engle & Kane, 2004; O’Donnell & Eggemeier, 1986). For example, processing an interviewer’s smile, eye gaze, or facial expressions may reduce the cognitive resources available for recalling or reporting the details of an event. One way we can reduce visual distractions in the environment and increase cognitive resources for the task is through inhibitory attentional mechanisms (Hasher et al., 1999, 2001)—these attentional mechanisms internally suppress goal-irrelevant features. Another more direct and perhaps less complex approach is to actively disengage from the environment by averting one’s gaze (Buchanan et al., 2014; Kleider-Offutt et al., 2016; Vredeveldt et al., 2011, 2014).

In several studies, researchers have explored the impact of the interviewer’s eyes and gaze on participants’ performance on cognitive tasks. For instance, Buchanan et al. (2014) examined the effects of eye contact and social gaze on participants’ performance on two cognitive tasks of increasing difficulty: mentally traversing through a 2D matrix (squares drawn in black ink on white cardboard) and then a 3D matrix (constructed with wooden blocks). In that study, 30 undergraduate students were randomly assigned to one of five conditions, with only one condition that involved reciprocated gaze: In the *eye contact* condition*,* eye contact was maintained between the interviewer and the participant. The other four conditions did not allow for reciprocated gaze: In the *mutual gaze* condition, participants gazed at the interviewer wearing a pair of dark glasses that obscured the interviewer’s eyes, in the *eye-closure* condition, participants closed their eyes, in the *gaze averted* condition*,* participants gazed toward the interviewer who did not reciprocate the gaze, and in the *head occluded* condition*,* participants gazed towards the interviewer who had occluded her face with a paper bag. Participants were instructed to mentally transverse through each matrix (e.g., to move up, down, left, right, forward, backward); directional instructions were presented at 0.5 s intervals. Task performance was measured by the number of matches and mismatches to the correct square (or box) on each instruction.

Overall, participants performed worse on the more difficult 3D task relative to the easier 2D task, and participants in the *eye contact* condition performed worse relative to the other four conditions on both cognitive tasks. To a lesser degree, in the *mutual gaze* condition, there was a decline in participants’ performance on the more difficult task compared with the three other nongaze conditions (*eye-closure* condition, *gaze averted* condition, and *head occluded* condition). The authors concluded that both eye contact and mutual gaze can disrupt cognitive task performance during face-to-face interactions due to the heightened cognitive demands associated with maintaining eye gaze. As such, disengaging eye gaze may provide additional benefits to performance on cognitively demanding tasks.

Glenberg et al. (1998) revealed similar results in their study. In that study, the authors randomly assigned 29 university students to either the *close eyes* condition or the *look* condition (gaze at the experimenter’s nose) while responding to a series of general knowledge questions and mathematical questions of increasing difficulty. The experimenter presented each question on a flash card for 10 seconds and then participants responded with their answers while engaged in condition-related behavior (i.e., eye closure or gaze at the experimenter’s nose). Participants responded to the questions with greater accuracy in the *close eyes* condition relative to the *look* condition for the moderate and difficult tasks only. These results suggest that, in addition to averting eye gaze, averting gaze from the interviewer’s nose may also promote better performance on cognitive tasks. Researchers have posited a phenomenon of ‘mind contact’ (Colombatto et al., 2019)—disruptions to cognitive task performance, including autobiographical recall and working memory during face-to-face interactions are not only specific to eye gaze, but this disruption can also occur when individuals gaze toward the examiner/interviewer’s nose (Glenberg et al., 1998) or mouth (Colombatto et al., 2019). Averting gaze to disengage from all facial features may facilitate internal control, thereby increasing cognitive task performance.

With this background in mind, we may be able to improve interview performance by inviting participants to close their eyes during an interview or by completely removing the interviewer’s face to reduce any visual communication cues associated with the interviewer. The advent of avatar technology has allowed for such flexibility in modifying the interviewer's characteristics. Avatars are computer-animated virtual characters^1^ that have been used in evidential interviewing studies. The use of avatars in the field of evidential interviewing largely falls into one of two categories: avatars as a tool to train interviewers or avatars as interviewers, per se. When avatars are used to train human interviewers, the trainees are typically given the opportunity to interview a child avatar who describes their experience of sexual abuse. One clear advantage of this approach is that interviewers can practice evidence-based interviewing techniques on an (avatar) child who reflects the experience and responses of a child sexual abuse victim (Kask et al., 2022; Pompedda et al., 2015, 2017, 2022).

In another, albeit small, body of work, researchers have provided promising data on the value of avatars as interviewers (Hsu & Teoh, 2017; Taylor & Dando, 2018). For example, Hsu and Teoh (2017) examined children’s reports of a prior event when a human interviewer or an avatar interviewed them. Prior to the interview, children participated in a treasure hunt activity that included some of the elements typically seen in sexual abuse cases (e.g., role play, taking photos). An avatar interviewer conducted the interviews based on the National Institute of Child Health and Human Development Investigative Interview Protocol (NICHD; Orbach et al., 2000). The avatar interviewer in that study was human operated and constructed using photorealistic eyes and lips with limited gestures (i.e., timed head tilts and eye blinks). Relative to being interviewed by the human interviewer, participants interviewed by the avatar interviewer provided more accurate responses to free recall questions, but not to specific questions.

In Taylor and Dando’s (2018) study, 38 adult participants watched a prerecorded video of a mock crime depicting the details of a car theft and subsequent events following the transgression. Two days later, participants were interviewed about the video using the UK investigative interview model known as PEACE (Ministry of Justice, 2011), which included best practice interview techniques. Either a human interviewer or an avatar interviewed independent groups of participants. In the avatar condition, both the participant and interviewer were presented as avatars in a 3D immersive, virtual environment; the participant and interviewer operated their respective avatars. The avatars’ facial features were computer generated, with no bodily movements. Taylor and Dando found that participants who were interviewed by the avatar provided more accurate responses to specific questions, but not to free-recall questions.

Taken together, in the two avatar studies described above, under certain conditions, avatar interviewers were better than human interviewers in eliciting complete and accurate accounts from adults and children. Given that both studies included best practice interviews, the authors hypothesized that the benefit of the avatar was that it presented fewer visual communication signals—gestures, facial expressions—that may have been potentially distracting to participants. Given this line of reasoning, we might predict that removing the interviewer’s face entirely might lead to even better performance. Although this kind of manipulation is difficult to achieve when using human interviewers, it can be easily achieved with an avatar.

## Impact of social evaluation on cognitive tasks

In the context of the broader framework of attentional control theory described above, participants’ perception of being social evaluated by someone watching them may hinder performance on cognitive tasks, particularly for more difficult tasks (Beilock et al., 2007; Oei et al., 2006). More specifically, when performing a cognitive task, such as providing a memory report, we need to devote our limited cognitive resources to the task at hand (Sweller et al., 1998). Under social-evaluative conditions (e.g., a sense of ‘being watched’), much of our cognitive resources may shift toward monitoring social-evaluative threat such as an interviewer’s nonverbal feedback, leaving little cognitive capacity for memory retrieval (Berggren & Derakshan, 2013; Eysenck & Derakshan, 2011). The influence of perceived social evaluation on memory performance follows the same basic idea of attentional interference described above.

The results of Buchanan et al.’s (2014) study described above illustrate the likely effects of social evaluation on cognitive task performance. Recall that in that study, participants were randomly assigned to one of five conditions to perform cognitive tasks, in only one condition—the *eye contact* condition—were participants engaged in reciprocal gaze (i.e., gaze towards the researcher and being watched by the researcher). The authors demonstrated that participants in this condition showed the worst performance on a cognitive task relative to the other four experimental conditions, suggesting that the addition of a social component may also hinder task performance (see also Doherty-Sneddon & Phelps, 2005).

## The present study

The overarching goal of the present study was twofold: (1) to first establish the efficacy of a human-appearing avatar (Exp 1) before comparing that avatar with the efficacy of a faceless avatar on adults’ memory reports (Exp 2) and (2) to examine participants’ understanding of the avatar’s ‘method of operation’ on their reports to investigate the impact of social evaluation ‘being watched’—associated with avatar interviewers. To create the sense of ‘being watched,’ in avatar studies, researchers have led participants to believe that the avatar they interacted with was either computer or human operated (Lucas et al., 2014). Participants watched a 5-min video and were subsequently interviewed about it either 1 day or 6 weeks later. The inclusion of a delay allowed us to test the effects of task difficulty on participants’ memory reports (Buchanan et al., 2014; Glenberg et al., 1998). We assumed that the longer retention interval would increase the cognitive demands associated with the memory interview (Glenberg et al., 1998). Furthermore, the inclusion of a longer retention interval improved the ecological validity of the present experiment because witnesses or victims are often interviewed following long delays (Hanna et al., 2010; Sutherland & Hayne, 2001).

In Experiment 1, we established the efficacy of a human-appearing avatar. Additionally, participants assigned to the avatar interviewer condition were asked whether they thought the interviewer was computer operated or human operated. In Experiment 2, we compared the efficacy of that human-appearing avatar with the efficacy of a faceless avatar. Additionally, we explicitly told participants whether the interviewer was computer operated or human operated.

## Experiment 1

In Experiment 1, we established the efficacy of our avatar interviewer by comparing the performance of participants who were interviewed by a human-appearing avatar interviewer to the performance of participants who were interviewed by a human interviewer.

### Method

#### Participants

Results from previous studies on eyewitness memory using stimuli similar to those used here demonstrated a medium effect size (Sutherland & Hayne, 2001). An a priori power analysis with a medium effect size of 0.30 (*f*) at an alpha level of .05 was conducted using G*Power 3.1 (Faul et al., 2007) to test the effects between interviewer type (avatar vs. human) and retention interval (1 day vs. 6 weeks) using a two-way analysis of variance (ANOVA). Power analysis revealed that a total sample of 90 participants was required to achieve a power of .80. Sample-size calculation with the same effect size and alpha level was conducted for the two one-sided test (TOST) to test the equivalence of memory performance as a function of interviewer type. Results showed that a total sample of 45 participants was required to achieve a power of .80. We recruited more participants (*N* = 108) than needed to accommodate for likely attrition due to a long retention interval between interviews. Two participants did not attend Session 2 of the experiment; the final sample consisted of 106 adults (88 females; *M*_age_ = 23.54 years; *SD* = 6.15; range: 18–59 years). Participants were recruited through a university participant database and reimbursed at local rates for their participation. The research received institutional ethical approval (D17/105).

#### Target event

The target event was depicted in a 5-min video (adapted from Sutherland & Hayne, 2001) that involved a female child, Sally, who is separated from her mother because she was distracted by a dog. In the video, Sally follows the man walking the dog and becomes lost. In the process, Sally also loses her toy monkey, which is then picked up by two delinquent teenagers—a boy and a girl—who tear the monkey apart. Sally is then approached by another man in the park who offers to help her find her toy monkey. Sally refuses this help, and the man drives away when he sees a policeman. The policeman asks Sally for her mother’s name, phone number, and where she works. The policeman takes Sally to the police station and calls her mother at work. Sally’s mother arrives at the police station and is reunited with Sally.

#### Interview conditions

Participants interacted face-to-face with either a human interviewer or an avatar interviewer that was displayed on a 21.5-inch computer monitor.

##### The human interviewer

**Fig. 1:**
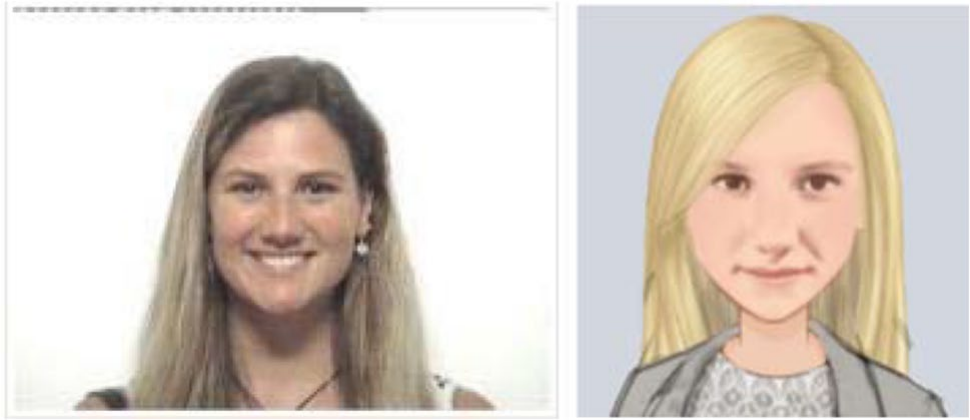
The human interviewer (left); the human-appearing avatar (right)

In the human-interviewer condition, participants were interviewed by a female human interviewer who was a final year undergraduate psychology student (see Fig. 1, left). The female interviewer sat across a small table from the participant.

##### The avatar interviewer

The human-appearing avatar interviewer was controlled using the “Wizard-of-Oz” technique—a technique where the avatar acts as an intermediary between the participant and a concealed human interviewer (Kelley, 1983). That is, the avatar is not computer controlled but rather is controlled by a human. This technique has been previously used in other avatar interviewing studies (e.g., Hsu & Teoh, 2017). In the present experiment, the same experimenter who performed the role as the human interviewer voiced and operated the avatar interviewer from a remote location. When the concealed human interviewer spoke, the human-appearing avatar’s lips moved synchronously with the human’s voice. The human-appearing avatar had photorealistic eyes and lips, and its eyes blinked and its head tilted to one side every 5 s in an alternating manner. The rate of blinking mimicked that of a typical human adult (Bentivoglio et al., 1997). The avatar was constructed to resemble the human interviewer in the experiment in facial features, hair color and style, and body shape (see Fig. 1, right).

#### Procedure

##### Session 1

At the beginning of the first session, participants were informed that the purpose of the experiment was to evaluate the suitability of a video for children. Each participant watched the video individually and afterward, they completed an online questionnaire to collect demographic information (gender, ethnicity, age, level of education).^2^ Participants were asked to return to the laboratory either 1 day or 6 weeks later.

##### Session 2

One day or 6 weeks after watching the video, participants returned to the laboratory and were told that the real purpose of the experiment was to assess their memory for the video. Participants were randomly assigned to one of two interviewer conditions: the human-interviewer condition or the avatar-interviewer condition. For participants in the human-interviewer condition, the experimenter introduced the female human interviewer by saying, “*This is Ana, and she is going to talk to you about the video.*” Participants in the avatar-interviewer condition were seated in front of a computer monitor that displayed the avatar interviewer. The experimenter then said, “*This is Ana. Ana is a computer animation that is going to talk to you about the video. You can talk to it, and it will respond*.” The experimenter then left the interview room.

The memory interview was conducted in two phases. During the free-recall phase, the interviewer asked the participant to report everything that he or she could remember from the video by saying, “*Tell me everything that you can remember about what happened in the video, from the beginning to the end*.” The interviewers were restricted to provide utterances only (e.g., “uh huh, hmm). The only additional prompt given during the free-recall phase was, “*Is there anything else you can remember that you haven’t already told me?”* Once the participant indicated that he or she had no more information to report, the interviewer began the direct-recall phase. The interviewer asked participants six direct-recall questions regarding specific aspects of the video (e.g., ‘What color was Sally’s monkey?’). Immediately after the interview, participants assigned to the avatar condition were asked if they thought that the avatar interviewer was operated by a computer or a concealed human.^3^

#### Coding and reliability

The interviews were all audio-recorded and transcribed verbatim.

##### Free-recall phase

4 To code the amount of correct information reported, participants received one point for each item of the information correctly reported in response to the general, open-ended question. For example, for the statement, “The little girl then saw a man with a small dog,” the participant would receive four points; one point each for the objects (i.e., the little girl, man, small dog) and one point for the action (i.e., saw). Repeated information was only coded the first time that it was mentioned. In instances where participants changed their responses, only their last response was coded. The same procedures were used to code errors.

To assess interobserver reliability, one observer coded all the free-recall accounts, and a second observer coded 25% of them. Neither observer was aware of the participants’ group assignments. A Pearson’s product-moment correlation yielded an interobserver reliability coefficient of *r* = .97, *p < .*001 for the total amount of information reported. Any discrepancies between the two observers were subsequently discussed and resolved.

##### Direct-recall phase

Participants received one point for each correct response to the six specific questions regarding specific aspects of the video. Again, to assess interobserver reliability, one observer coded all the participants’ responses during the direct-recall phase, and a second observer coded 25% of them. There was no discrepancy in the coding of the answers to the six direct questions.

### Results and discussion

#### Preliminary analyses

We carried out an exploratory analysis of the dependent variables (amount of correct information, accuracy) to identify any outlier data points. One extreme outlier data point was removed from all the subsequent analyses (*N* = 105); this participant reported having no recollection of watching the target video. This participant was in the avatar condition and had been interviewed after the 6-week delay. Any other outliers were included in the analysis because a careful inspection of the 5% trimmed mean showed that these scores did not have a strong influence on the mean. The descriptive data for participants in each experimental condition are provided in Table 1.

**Table 1:** Descriptive data of participants in each experimental condition

Retention interval	Interviewer type			
	Avatar		Human	
	1 Day (*n* = 27)	6 Week (*n* = 26)	1 Day (*n* = 27)	6 Week (*n* = 25)
Age [Mean (***SD***)]	23.26 (7.58)	22.62 (3.63)	25.67 (7.81)	22.64 (3.99)
Gender (M:F:Other)	3:24:0	4:22:0	5:22:0	6:19:0

#### Memory interview

##### Free-recall phase

**Fig. 2:**
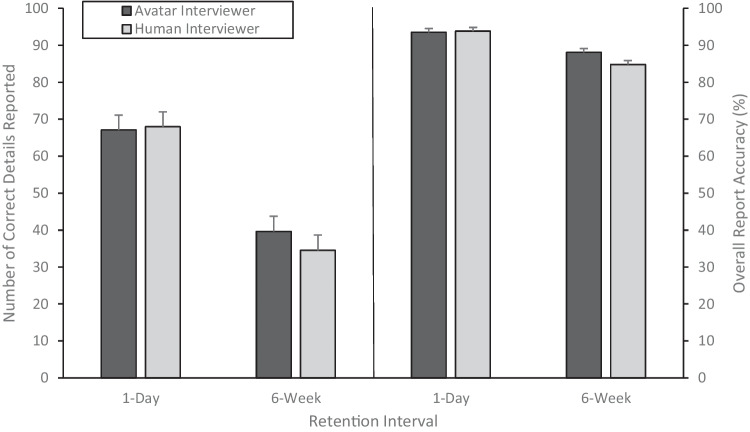
Left panel: The amount of correct information (+1SE) reported during free recall. Right panel: Overall accuracy (%) of information (+1SE) reported by participants during free recall

We measured memory performance during free recall using the amount of correct information reported, the amount of incorrect information reported, and the accuracy of participants’ free-recall accounts (correct information/total information). The memory performance data were submitted to a 2 (interviewer type) × 2 (retention interval) ANOVA. As shown in Fig. 2 (left panel), there was a main effect of retention interval; participants in the 1-day retention group reported almost twice as much correct information (*M*_1d retention_ = 67.54, *SD* = 25.23) than did participants in the 6-week retention group (*M*_6w retention_ = 37.12, *SD* = 14.96), *F*(1, 101) = 55.14, *p* < .001, ${\upeta}_p^2$ = .35. We found no statistically significant difference in the amount of correct information reported as a function of interviewer (*M*_avatar_ = 53.62, *SD* = 26.70; *M*_human_ = 51.88, *SD* = 25.07), *F*(1, 101) = 0.27, *p* = .60, ${\upeta}_p^2$ = .003, and no interaction, *F*(1, 101) = .53, *p* = .47, ${\upeta}_p^2$ = .005. There was also no statistically significant difference in the amount of incorrect information reported as a function of interviewer (*M*_avatar_ = 5.26, *SD* = 4.06; *M*_human_ = 5.46, *SD* = 3.42 ), *F*(1, 101) = 0.09, *p* = .76, ${\upeta}_p^2$ = .001, nor retention interval, (*M*_1d retention_ = 4.80, *SD* = 3.46; *M*_6w retention_ = 5.96, *SD* = 4.07), *F*(1, 101) = 2.57, *p* = 0.11, ${\upeta}_p^2$ = .03; and no interaction, *F*(1, 101) = 0.31, *p* = .58, ${\upeta}_p^2$ = .003.

To obtain an accuracy score for each participant, we divided the total amount of correct information reported during free recall by the total amount of information reported (i.e., correct information + incorrect information). The data shown in Fig. 2 (right panel) were submitted to a 2 (interviewer) × 2 (retention interval) ANOVA. As shown in Fig. 2 (right panel), there was a main effect of retention interval; participants in the 1-day retention group provided more accurate reports (*M =* 93.67%*, SD* = 3.45) than did participants in the 6-week retention group (*M =* 86.50%, *SD* = 6.77), *F*(1, 101) = 49.30, *p <* .001*,* ${\upeta}_p^2$ = .33. We found no statistically significant difference in accuracy as a function of interviewer (*M*_avatar_ = 90.85%, *SD* = 6.48; *M*_human_= 89.51%, *SD* = 6.33), *F*(1, 101) = 2.04, *p* = .16, ${\upeta}_p^2$ = .02, and no interaction, *F*(1, 101) = 3.03, *p* = .09, ${\upeta}_p^2$ = .03.

##### Direct-recall phase

The number of correct responses to the six direct questions were submitted to a 2 (interviewer) × 2 (retention interval) ANOVA. There was a main effect of retention interval; participants in the 1-day retention group correctly answered a greater number of questions (*M* = 3.69, *SD* = .89) than did participants in the 6-week retention group (*M =* 2.26, *SD* = 1.09), *F* (1, 101) = 54.23, *p* < .001, ${\upeta}_p^2$ = .35. Once again, we did not find an effect of interviewer (*M*_avatar_ = 3.02, *SD* = 1.15; *M*_human_ = 2.96, *SD* = 1.30), *F*(1, 101) = 0.16, *p* = .69, ${\upeta}_p^2$ = .002, and no interaction, *F*(1, 101) = 0.93, *p* = .34, ${\upeta}_p^2$ = .001.

**Fig. 3:**
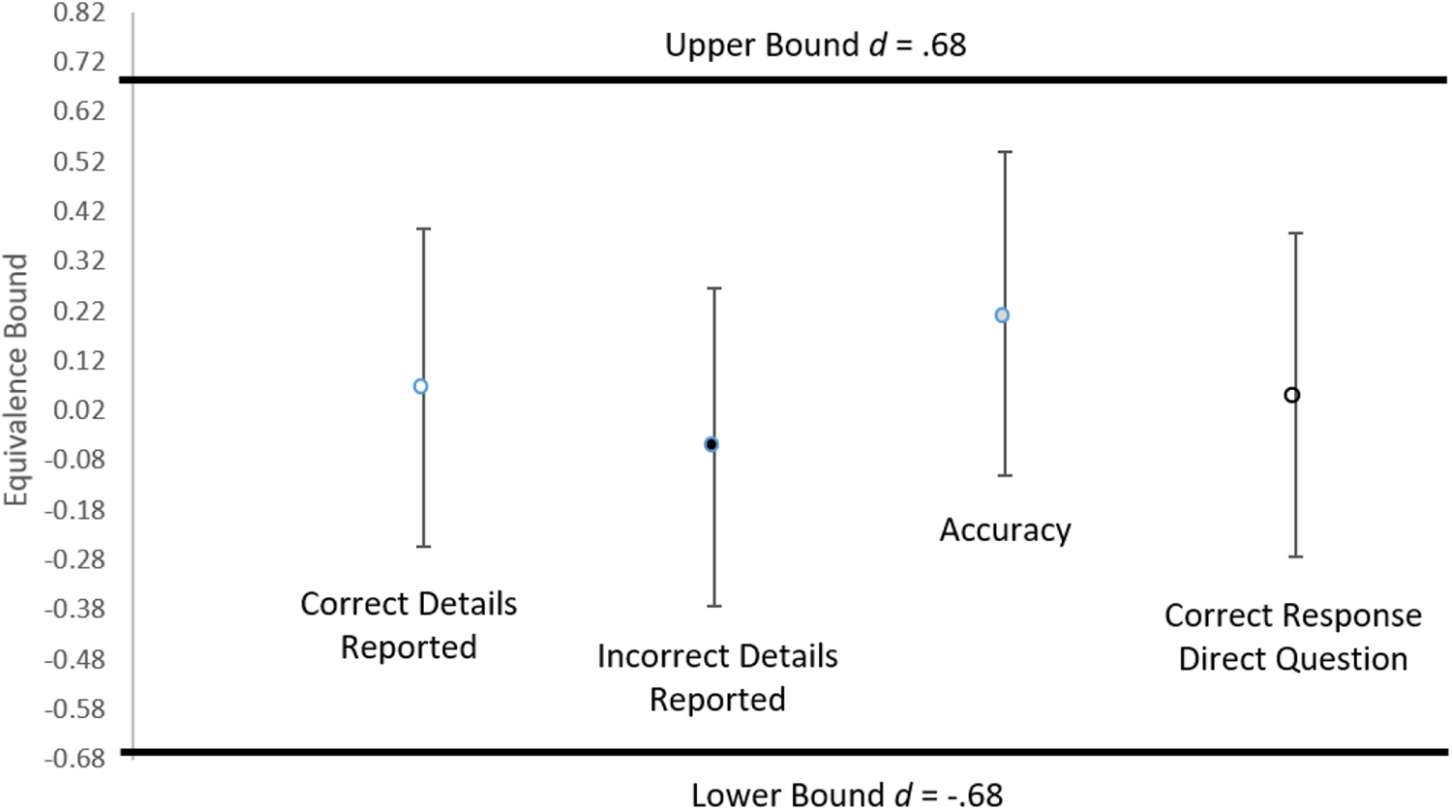
Confidence interval (90% CI) of memory performance data as a function of interviewer type

To further test for equivalence in the participants’ memory performance as a function of interviewer, we conducted TOST (Schuirmann, 1987). An equivalence bound of *d* = −0.68 to *d* = 0.68 were derived from the critical *F* value (4.11; Lakens et al., 2018) obtained from a previous avatar investigative interviewer study with adults (Taylor & Dando, 2018). As shown in Fig. 3, TOST revealed statistical equivalence in participants’ memory performance when an avatar or human interviewed them—correct details reported, *t*(103) = −3.14, *p* = .001; incorrect details reported, *t*(103) = −3.75, *p* < .001; accuracy, *t*(103) = −2.41, *p* = .009; correct responses to direct questions, *t*(103) = −3.24, *p* < .001.

##### Understanding of control

Next, we turned our focus to participants in the avatar condition (*n* = 53) who were asked about the avatar interviewer’s method of operation (computer or human operated). Participants were assigned to one of two operation groups: the computer-operation group (*n =* 14) if they indicated that the avatar was controlled by a computer system, or the human-operation group (*n =* 39) if they indicated that a concealed human controlled the avatar.

A 2 (operation group) × 2 (retention interval) ANOVA indicated that there was no effect of operation group on the amount of correct information reported (*M*_avatar-operated_ = 50.43, *SD* = 18.52; *M*_human-operated_ = 54.77, *SD* = 29.21), *F*(1, 49) = 0.01, *p* = .92, ${\upeta}_p^2$ < .001; the amount of incorrect information reported (*M*_avatar-operated_ = 4.07, *SD* = 4.10; *M*_human-operated_ = 5.69, *SD* = 4.01), *F*(1, 49) = 1.96, *p* = .17, ${\upeta}_p^2$ = .04; or on the number of correct responses to the direct questions (*M*_avatar-operated_ = 2.64, *SD* = 1.45; *M*_human-operated_ = 3.15, *SD* = 1.01), *F*(1, 49) = 0.92, *p* = .34, ${\upeta}_p^2$ = .02, There was, however, a main effect of operation group on accuracy during free recall. Participants who indicated that the avatar interviewer was computer-operated provided free-recall accounts with greater accuracy (*M* = 93.57%, *SD* = 5.22) than did participants who had indicated that the avatar interviewer was human-operated (*M* = 89.88%, *SD* = 6.66), *F*(1, 49) = 6.43, *p* = .01, ${\upeta}_p^2$ = .12.^5^

In summary, in Experiment 1, we found equivalence in the amount and accuracy of the information that participants reported as a function of interviewer. That is, participants interviewed by the human-appearing avatar performed equivalently to the participants interviewed by the human interviewer. This finding paves the way for a comparison in Experiment 2 between the efficacy of a human-appearing avatar interviewer (equivalent to a human interviewer) and the efficacy of a faceless avatar. In Experiment 1, although there was no impact of the interviewer, there was an impact of the participants’ perception of the avatar’s method of control. That is, participants who indicated that the avatar interviewer was computer operated provided more accurate details of the target video than did the participants who indicated that the avatar was human operated. This finding suggests that, in addition to the impact of an avatar interviewer's facial features, participants’ perception of the avatar’s operation may also influence performance. One caveat to the finding was the unequal sample size between the two operation groups, resulting in an underpowered analysis. In Experiment 2, we controlled for sample size across the two experimental conditions by explicitly telling participants that the interviewer was either computer operated or human operated.

## Experiment 2

Based on our findings in Experiment 1 of equivalence in participants’ memory performance when a human interviewer or human-appearing avatar interviewed them, in Experiment 2, we compared the efficacy of a human-appearing avatar with the efficacy of a faceless avatar. A faceless avatar has no visual communication cues that may distract participants during recall, which we hypothesized would promote better memory performance.

### Method

#### Participants

An a priori power analysis with a medium effect size of 0.30 (*f*) at an alpha level of .05 was conducted using G*Power 3.1 (Faul et al., 2007) to test the effects between interviewer group (human-appearing avatar, faceless avatar) and informed agency (human operated, computer operated) using a two-way ANOVA. Power analysis revealed that a total sample of 90 participants was required to achieve a power of .80. Again, we recruited more participants (*N* = 119) than were needed to accommodate for likely attrition due to a long retention interval between interviews. Eight participants failed to return to Session 2 of the experiment so our final sample included 111 participants (88 females; *M*_age_ = 22.40 years; *SD* = 5.83; range: 18–60 years). Participants were recruited through a university participant database and reimbursed at local rates for their participation. The experiment received institutional ethical approval.

#### Interview conditions

Participants interacted with one of two avatar interviewers who were displayed on a 21.5-inch computer monitor placed on a table in front of the participant.

##### The human-appearing avatar

The human-appearing avatar in Experiment 2 was identical to the human-appearing avatar in Experiment 1.

##### The faceless avatar

**Fig. 4:**
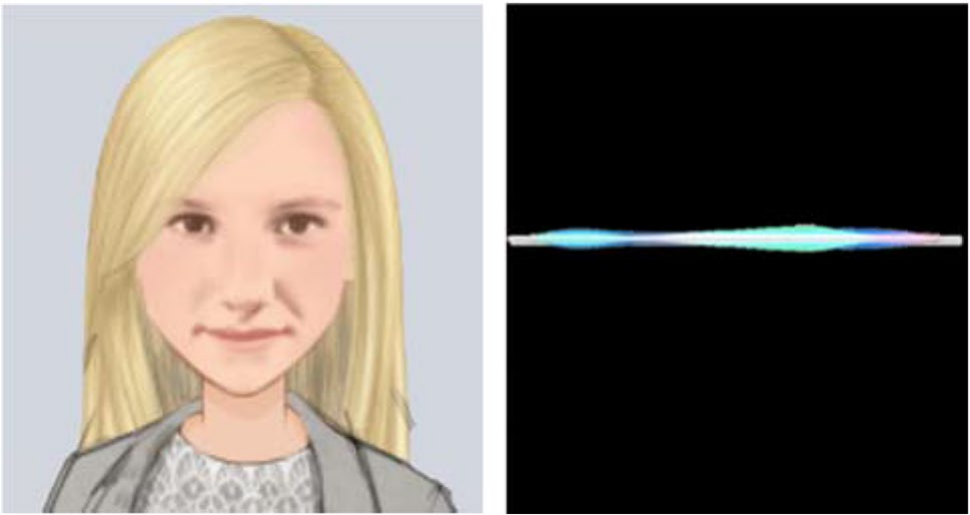
The human-appearing avatar interviewer (left); the faceless avatar interviewer (right)

The faceless avatar interviewer was depicted as a speech-wave pattern (see Fig. 4). The same concealed human interviewer who operated the human-appearing avatar also operated the faceless avatar interviewer and provided its voice from another room via the internet using the Wizard-of-Oz technique. When the concealed human interviewer spoke, the speech wave moved up and down in synchrony with the human voice.

#### Procedure

##### Session 1

The same target video used in Experiment 1 was used in Experiment 2.

##### Session 2

In Experiment 2, all participants were interviewed 6 weeks after viewing the video. Participants were randomly assigned to one of the two interviewer conditions: the human-appearing avatar condition (*n* = 55) or the faceless-avatar condition (*n* = 56). Half of the participants in each interview condition were randomly assigned to one of two informed agency conditions: the informed-human condition or the informed-computer condition. For participants in the informed-human condition, the experimenter introduced the interviewer by saying, “*This is Ana, a computer animation that works like a puppet. It allows a person in another room to have a conversation with you. My colleague will be sitting in the other room and will be able to see and hear you on this screen. She has access to a set of prerecorded questions and responses that will be used to have a conversation with you about the video. You can start the conversation by saying, Hello*” (adapted from Lucas et al., 2014).

For participants in the informed-computer condition, the experimenter introduced the interviewer by saying, “*This is Ana, a computer animation that uses artificial intelligence to have a conversation with you about the video. The system gets audio and visual input from you. It uses a speech recognition tool to understand what you’re saying, then uses a complex series of equations to choose the best way to respond. You can start the conversation by saying, Hello*” (adapted from Lucas et al., 2014).

The memory interview in Experiment 2 was identical to that in Experiment 1: Participants were initially asked for a free-recall account and then they were asked specific questions during the direct-recall phase of the interview.

#### Coding and reliability

The interviews were all audio-recorded, transcribed verbatim, and coded using the same coding scheme as in Experiment 1. A Pearson’s product-moment correlation yielded an interobserver reliability coefficient of *r* = .98, *p < .*001 for the total amount of information reported in free recall. Any discrepancies between the two observers were subsequently discussed and resolved. There was no discrepancy in the coding of the answers to the six direct-recall questions.

### Results and discussion

#### Preliminary analysis

In the first part of the analysis, we carried out an exploratory analysis of the dependent variables (amount of correct information, accuracy) to identify any outlier data points. Two participants in the faceless-avatar condition were identified as statistical outliers and were excluded in subsequent analyses (*N* = 109); any other outliers were included in the analysis because a careful inspection of the 5% trimmed mean showed that these scores did not have a strong influence on the mean. The descriptive data for participants in each experimental condition are provided in Table 2.

**Table 2:** Descriptive data of participants in each experimental condition

Informed agency	Avatar interviewer type			
	Human-appearing		Faceless	
	Computer operated (*n* = 27)	Human operated (*n* = 28)	Computer operated (*n* = 27)	Human operated (*n* = 27)
Age [Mean(*SD*)]	23.44 (8.71)	20.75 (2.85)	21.56 (3.46)	23.93 (6.45)
Gender (M:F:Other)	6:21:0	3:25:0	6:21:0	8:18:1

#### Memory interview

##### Free-recall phase

**Fig. 5:**
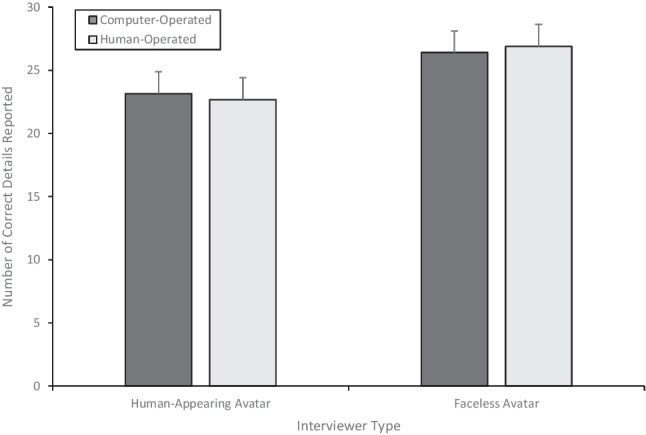
The amount of correct information (+1SE) reported by participants during free recall

We measured memory performance using the amount of correct information reported (see Fig. 5), incorrect information reported, and the accuracy of participants’ free-recall accounts. The memory performance data were submitted to a 2 (interviewer) × 2 (informed agency) ANOVA. As shown in Fig. 5, there was a main effect of interviewer; participants who were interviewed by the faceless avatar reported significantly more correct information (*M*_faceless avatar_ = 26.65, *SD* = 10.15) than did participants who were interviewed by the human-appearing avatar (*M*_human-appearing avatar_ = 22.91, *SD* = 7.65), *F*(1, 105) = 4.63, *p* = .03*,* ${\upeta}_p^2$ = .04. We did not find an effect of informed agency (*M*_Informed Computer_ *M* = 24.78, *SD* = 8.80; *M*_Informed Human_ = 24.75, *SD* = 9.52), *F*(1, 105) < .001, *p* = 1.00, ${\upeta}_p^2$ < .001, nor an interaction, *F*(1, 105) = 0.08, *p* = .79, ${\upeta}_p^2$ = .001.

For incorrect information reported, there was also a main effect of interviewer; participants who were interviewed by the faceless avatar reported significantly more incorrect information (*M*_faceless avatar_ = 3.19, *SD* = 1.94) than did participants who were interviewed by the human-appearing avatar (*M*_human-appearing avatar_ = 2.42, *SD* = 1.57), *F*(1, 105) = 5.09, *p* = .03*,* ${\upeta}_p^2$ = .05. We did not find an effect of informed agency (*M*_Informed Computer_ = 2.78, *SD* = 1.63; *M*_Informed Human_ = 2.82, *SD* = 1.96), *F*(1, 105) = .02, *p* = .88, ${\upeta}_p^2$ < .001, nor an interaction, *F*(1, 105) = 1.90, *p* = .17, ${\upeta}_p^2$ = .02.

To obtain an accuracy score for each participant, we divided the total amount of correct information reported during free recall by the total amount of information reported (i.e., correct information + incorrect information). The accuracy scores were submitted to a 2 (interviewer) × 2 (informed agency) ANOVA. Overall, accuracy was high (89.97%) and we did not find a statistically significant difference in accuracy as a function of interviewer (*M*_human-appearing avatar_ = 90.15%, *SD* = 6.44%; *M*_faceless avatar_ = 89.79%, *SD* = 5.18%), *F*(1, 105) = 0.09, *p* = .76, ${\upeta}_p^2$ = .001, nor informed agency (*M*_Informed Human_ = 90.20%, *SD* = 6.02%; *M*_Informed Computer_ = 89.73%, *SD* = 5.67%), *F*(1, 105) = 0.16, *p* = .69, ${\upeta}_p^2$ = .002, and no interaction, *F*(1, 105) = 1.46, *p* = .23, ${\upeta}_p^2$ = .01.

##### Direct-recall phase

Next, we examined participants’ memory performance when they were asked six direct-recall questions. A 2 (interviewer) × 2 (informed agency) ANOVA revealed no statistically significant difference in the number of correct responses to the six direct-recall questions as a function of interviewer (*M*_human-appearing avatar_ = 2.42, *SD* = 1.27; *M*_faceless avatar_ = 2.50, *SD* = 1.13), *F*(1, 105) = 0.12, *p* = .74, ${\upeta}_p^2$ = .001 or informed agency (*M*_Informed Human_ = 2.31, *SD* = 1.17; *M*_Informed Computer_ = 2.61, *SD* = 1.22), *F*(1, 105) = 1.70, *p* = .20, ${\upeta}_p^2$ = .02, and no interaction, *F*(1, 105) = 0.25, *p* = .62, ${\upeta}_p^2$ = .002.

In summary, the results of Experiment 2 demonstrated that participants reported more correct information (but also more incorrect information) when the faceless avatar interviewed them as opposed to a human-appearing avatar. In Experiment 2, we did not find any impact of the information given to participants about the avatar’s method of control on the quality of participants’ memory reports.

## General discussion

The overarching goal of the present study was twofold: (1) to first establish the efficacy of a human-appearing avatar (Experiment 1) before comparing that avatar with the efficacy of a faceless avatar on adults’ memory reports of a prior event (Experiment 2) and (2) to evaluate the effects of social evaluation, specifically ‘being watched’ on participants’ reports. In Experiment 2, we found that, relative to participants who were interviewed by a human-appearing avatar (which based on our findings in Experiment 1 is equivalent to being interviewed by a human interviewer face-to-face), participants reported more correct information (but also more incorrect information) when the faceless avatar interviewed them. There was no influence of the information received about the avatar’s method of operation on memory reports.

While our data show that the absolute difference in errors was less than one piece of information (i.e., incorrect details reported: *M*_human-appearing avatar_ = 2.42 versus *M*_faceless avatar_ = 3.19), the effect sizes suggest that this small difference in errors may still have practical significance (Otgaar et al., 2022). From a theoretical standpoint, this finding challenges our hypothesis that a faceless avatar regulates attentional control and reduces cognitive load to facilitate memory reports. If indeed the faceless avatar reduced the cognitive load during recall, then we would expect an overall improvement in accuracy (Camos & Portrat, 2015). Our findings, however, favor the notion that a faceless avatar may influence participants’ reporting criterion. That is, by removing important visual feedback from the avatar interviewer (i.e., facial features), we also diminished the social nature of the interview, thereby encouraging reporting without actively monitoring for accuracy (Goldsmith et al., 2002; Koriat & Goldsmith, 1996; see Gabbert et al., 2009; Gawrylowicz et al., 2014, for these similar findings from studies on self-administered interviews—an interviewing method that also removes the presence of the interviewer).

Related to this interpretation is the impact of a faceless avatar on people’s willingness or motivation to provide information. Researchers who have examined the impact of an avatars’ appearance on participants’ willingness to disclose personal details and embarrassing personal events have reported that, relative to human-appearing avatars, an avatar without facial features promoted disclosure of personal details or embarrassing personal events more so than did an avatar with human facial features (Hsu et al., 2023; Lind et al., 2013) or a video-conferencing interview (Bailenson et al., 2006). These results support the idea that a faceless avatar may increase participants’ willingness or motivation to report information, possibly over and above reducing distractions to produce quality memory reports.

In the present study, we also investigated the impact of participants’ interpretation of the avatar’s method of operation (computer or human operated). We inferred that participants who thought or received information that the avatar was human operated would have an elevated sense of “being watched” (Lucas et al., 2014), which may hinder the quality of memory reports. In Experiment 1, we found that adults who indicated that they thought that the avatar interviewer was computer operated provided more accurate details of the target video than did the participants who believed the avatar was human-operated. In our better-powered Experiment 2, we *explicitly told* participants that the interviewer was either computer operated or human operated. Under these conditions, we found that the information that participants received about the avatar’s method of operation did not affect their memory performance, suggesting that the finding from Experiment 1 may be subject to a false-positive effect. A limitation to Experiment 2 was that we did not directly measure the level that participants sensed that they were ‘being watched.’ Given the social nature of evidential interviews (both with human and avatar interviewers), participants’ willingness to report (or abstain) information under social-evaluative conditions should be considered in concert with the effects of cognitive load. Indeed, in some avatar studies, participants who were led to believe that an avatar interviewer was computer operated reported more willingness to disclose information and less intent to provide socially desirable information (Lucas et al., 2014; cf. Ho et al., 2018; Hsu et al., 2021). Future works should provide a direct measure of social-evaluative threat under different social conditions.

The results of the present study raise questions for future research. For example, in Experiment 2, we removed all visual features of a human from the avatar interviewer which limits our ability to ascertain whether it is particular human characteristics—eyes, nose, mouth, face, or overall human form—that influence memory performance. In subsequent research, the human characteristics of the avatar interviewer could be systematically removed to determine their influence on adults’ memory reports. In addition, we conducted the interviews in a controlled environment using open-ended and direct questions that were based on best practice methods to enhance recall. In a more natural setting, evidential interviews are typically long and complex, and interviewers may inadvertently incorporate leading and misleading questions that may alter the interviewee’s memory report. In a previous avatar interviewer study with children, Hsu and Teoh (2017) demonstrated a reduction in the accuracy of children’s memory report when an avatar interviewer (vs. a human) asked misleading questions. In the future, it would be important to examine the effectiveness of avatar interviewers under less optimal interviewing conditions. Finally, in the present study, we included a 1-day or 6-week retention interval between the witnessed event and recall of that event. In a legal context, there is usually a much longer delay between an allegation and subsequent police interviews (Hanna et al., 2010; London et al., 2005, 2008). In future research, we could also examine the impact of avatar interviewers when participants are interviewed after longer retention intervals.

## Conclusion

The main finding from the current study demonstrated that a faceless avatar elicited more correct (and incorrect) details of a prior event, suggesting that the benefit of this avatar could be to encourage reporting of information and improve quantity rather than quality of memory reports. The amount of incorrect information reported, however, may have little practical implications in court. In this sense, a faceless avatar interviewer may still be beneficial in a legal setting, as it could complement a human interviewer in eliciting large amounts of information under time-critical situations without much increase in error. The application of a faceless avatar may be similar to that of a self-administered interview but with the added benefit of a potentially more engaging visual interface. The current study paves the way for future scholars to design, test, and incorporate avatars as the future of evidential interviewing.

## Footnotes

1 In the present study, we refer to avatars as computer-animated objects that can be either computer or human operated (see Fox et al., 2015, for a definition of avatars and agents).

2 As a part of a larger study to examine individual variations in response to avatar interviewers, all participants completed the NEO-Five-Factor Inventory-3 (Costa & McCrae, 1992) to measure their general domains of personality. Results are to be reported elsewhere.

3 Participants in both interviewer conditions completed an 11-item feedback questionnaire. The questionnaire was designed to evaluate participants' thoughts on the interviewer, particularly around questions of disclosing embarrassing/sensitive information, and the results are to be reported elsewhere.

4 As part of a study on information disclosure, we coded for and analyzed the number of words that participants said during free recall, regardless of the content, as a measure of verbal interaction.

5 Consistent with the earlier analyses across the whole sample, within the avatar condition alone, there was a main effect of retention interval on the amount of correct information reported, *F*(1, 49) = 8.80, *p* = .005, ${\upeta}_p^2$ = .15, on accuracy during free recall, *F*(1, 49) = 7.59, *p* = .008, ${\upeta}_p^2$ = .13, and on the number of correct responses to the direct questions, *F*(1, 49) = 11.88, *p* = .001, ${\upeta}_p^2$ = .01. That is, participants in the 1-day retention group reported a greater amount of correct information (*M* = 67.11, *SD* = 26.75) and information with higher accuracy during free recall (*M* = 93.51%, *SD* = 3.74), and correctly answered a greater number of questions in response to the direct questions (*M* = 3.63, *SD* = .69) compared with participants in the 6-week retention group [amount of correct information *M* = 35.82, *SD* = 17.66; accuracy *M* = 88.09%, *SD* = 7.56; number of correct responses to direct questions *M* = 2.39, *SD* = 1.20]. There were no interaction effects for any of the dependent measures.
